# MEETING DATA COLLECTION GOALS QUICKER: AN EXPERIMENTAL EVALUATION TO REDUCE FIELDWORK DURATION IN A MIXED-MODE PANEL STUDY

**DOI:** 10.1093/jssam/smaf030

**Published:** 2026-01-08

**Authors:** KATHERINE A. MCGONAGLE, NARAYAN SASTRY

**Affiliations:** Research Professor with the Institute for Social Research, University of Michigan, P.O. Box 1248, Ann Arbor, MI 48104-1248, USA.; Research Professor with the Institute for Social Research, University of Michigan, Ann Arbor, MI, USA

**Keywords:** Fieldwork duration, Mixed-mode, Panel surveys, Response rates, Social psychology, Survey costs

## Abstract

An experiment was implemented in the 2023 wave of a US household panel study to assess the effects of a shortened field period on data collection outcomes. Following the recent adoption of sequential mixed-mode designs by panel studies worldwide, it has been observed that interview completion for respondents offered the initial mode of web is faster compared to those initially offered the telephone. This study describes an experiment designed to evaluate whether the new mixed-mode designs can support an accelerated field period and achieve cost savings while still meeting fieldwork goals. We assessed a shorter field period of 20weeks against the standard 28-week field period and randomized study participants to each condition. The treatment group received accelerated fieldwork protocols over a 20-week data collection period, and a control group received the same protocols over the standard 28-week period. We compare the effect of the shortened duration on fieldwork outcomes, including response rates, sample composition, interviewer effort, time to interview completion, survey costs, and interview quality. We find that the accelerated protocol yields higher response rates, lower interviewer effort, and cost savings with no differences in sample composition or decrements to interview quality. We describe the strengths and limitations of the study and provide suggestions for future research on fieldwork duration.

## 1. INTRODUCTION AND BACKGROUND

Panel studies worldwide are adopting sequential mixed-mode designs that prioritize the use of web, followed by the option of telephone or in-person interviewing as a strategy to maximize response rates and reduce survey costs. Among the national panel studies that have shifted to sequential web-first mixed-mode designs, several have conducted empirical evaluations and have generally found positive effects on various fieldwork outcomes, including response rates, sample composition, interviewer effort and survey costs (e.g., Jäckle, Lynn, and Burton, 2015; Bianchi et al. 2017; Jäckle, Gaia, and Benzeval, 2017; Brown and Calderwood 2020; Biemer et al. 2022; Voorpostel, Lipps, and Roberts, 2021; Sastry and McGonagle 2022; McGonagle and Sastry 2023). Following the successful transition to mixed-mode designs, research is now focused on enhancements to survey design features and contact protocols to improve fieldwork outcomes in this new setting. Some of these research topics include exploring incentive strategies (McGonagle, Sastry, and Freedman 2023; Booth et al. 2024; Cabrera-Álvarez and Lynn 2025), techniques for contacting respondents via text, email, and post mail (Cernat and Lynn 2018; Patrick et al. 2022; Zhang et al. 2023; Cabrera-Álvarez and Lynn 2024), the timing of contact attempts (Lynn, Bianchi, and Gaia 2024), optimizing the length of questionnaire text (Rettig and Blom 2024), the use of web-optimized devices like smartphones (Patrick et al. 2022), and more. One important gap in the assessment of mixed-mode survey design features is the evaluation of fieldwork outcomes for shortened fieldwork durations that capitalize on the substantially quicker pace of completing web interviews.

Achieving high panel response rates in an interviewer-administered environment has historically required lengthy data collection periods over which declining numbers of interviews are completed each week until a response rate goal is met (McGonagle et al. 2023; Minderop 2023). Interviews obtained in the later phases of fieldwork are comparatively expensive and contribute inordinately to the cost of data collection (Sastry and McGonagle 2022; Calderwood et al. 2023; McGonagle et al. 2023; Lynn, Bianchi, and Gaia 2024) but may be necessary to mitigate nonresponse bias (Struminskaya and Gummer 2022). In contrast to survey designs that rely primarily on interviewer administration, a key advantage of mixed-mode designs is that, on average, self-administered interviews occur earlier in the field period and are completed more quickly overall (Sastry and McGonagle 2022; Gummer and Struminskaya 2023; McGonagle and Sastry 2023).

A main benefit of the faster pace of self-administered interview completion is the potential for cost savings due to the need for fewer resources devoted to overall fieldwork staffing and management, including effort from field supervisors, managers, and interviewer staff. Moreover, a shorter interview period may provide greater standardization of interview responses by reducing the effects of seasonality and external shocks and events on the responses of sample members. By reducing the period over which contact attempts and requests for participation are made, respondents may experience less survey fatigue. Specifically, in the context of panel studies where fieldwork may last many months, reducing this period may decrease respondent annoyance and burden. Furthermore, a faster pace of data collection may help survey organizations process and release survey data more promptly to the scientific user community, ensuring the information is timely and relevant.

Despite these advantages, the impact on fieldwork outcomes of shortening the data collection period in the context of mixed-mode panel study designs is not known. Although the research evidence is very sparse, several potential risks of reducing fieldwork duration can be identified. With the backdrop of the well-documented finding that it is increasingly difficult to contact respondents (e.g., National Research Council, 2013; Beullens et al. 2018; de Leeuw et al. 2018; Williams and Brick 2018), a shorter duration may mean less time to successfully contact and persuade respondents to participate, both factors which may produce refusal outcomes (Groves and Couper 1998) and lead to panel attrition. Moreover, given prior findings that different groups of respondents may participate early or late in fieldwork (Lugtig 2014; Gummer and Struminskaya 2023; Minderop and Weiß 2023), other risks of a shorter field period are the potential for higher rates of item nonresponse (e.g., Olson 2013) and nonresponse bias (Sztabiński, Sztabiński, and Przybysz 2009; Struminskaya and Gummer 2022). In addition, while the body of research is scant on this issue, a shorter field period could yield unusually short interviews (Couper and Kreuter 2013) if respondents have less time to complete interviews they may have started.

Only a handful of studies have examined the topic of fieldwork duration in panel studies, and we know of no studies that have used an experimental design to manipulate fieldwork length and assess the impact on fieldwork outcomes. Previous studies have primarily conducted non-experimental evaluations of data collection outcomes for respondents who completed interviews at various time points during the fieldwork period (e.g., Lugtig 2014; Sigman et al. 2014; Minderop and Weiß 2023). For example, research has shown that panel attrition is higher for habitually late respondents (Minderop and Weiß 2023) and those with a variable response-time pattern (e.g., early response one wave, late response another wave; Lugtig 2014; Minderop and Weiß 2023). Other research has described the individual socio-demographic characteristics of respondents who participate early or late. For instance, a web-based survey of federal workers found early response was significantly higher among older, female, nonminority employees (Sigman et al. 2014). Respondents who participate late in fieldwork may also have poorer response quality in the form of shorter interview lengths (Olson and Smyth 2015; Kirchner and Olson 2017; Pirralha et al. 2024) and higher rates of item nonresponse (Olson 2013; Rao and Pennington 2013).

Social psychological and behavioral theories of decision-making applied to survey participation may provide insights into how shortening fieldwork duration affects respondent behavior. The leverage-saliency theory of survey participation suggests that individuals decide whether to participate in a survey based on how desirable they find various design features and the saliency of these features (Groves, Singer, and Corning 2000). For instance, individuals may be motivated to cooperate with a survey request due to perceived benefits such as the promised incentive, interest in the survey topic, loyalty to the study, or a desire to contribute to scientific research (Keusch 2015). In the context of an established panel study, shortening the deadline could motivate respondents to comply promptly in order to gain these perceived benefits before they expire. This prediction aligns with the scarcity principle of persuasion which posits that perceptions of desirability and compliance increase when options are limited or temporarily available (Groves, Cialdini and Couper 1992; Cialdini 1993; Cialdini and Trost 1998; Ariely and Zakay 2001), and the theory of regret-avoidance which suggests that individuals tend to behave in ways that minimize potential regret to optimize the outcomes they perceive as desirable (Pieters and Zeelenberg 2007; Zeelenberg and Pieters 2007).

In this article, we present findings from an experiment aimed at evaluating the impact of a shortened fieldwork duration on data collection outcomes in a long-standing, nationally representative household panel study that has recently transitioned to a sequential mixed-mode design. We assessed a shorter field period of 20weeks against the standard 28-week field period and randomized study participants to each condition. The treatment group received accelerated fieldwork protocols over a 20-week data collection period, and a control group received the same protocols over the standard 28-week period. Our main goal is to evaluate whether the new mixed-mode design can support an accelerated field period and achieve cost savings while still meeting fieldwork goals. We examine the effects of the shortened field length on a key set of outcome variables, including response rates, sample composition, interviewer effort and time to interview completion, survey costs, and response quality. To our knowledge, this is the first randomized experimental assessment of the effects of fieldwork length in a mixed-mode panel context and provides new empirical evidence on the optimal use of fieldwork protocols.

## 2. RESEARCH QUESTIONS

We examine four research questions:

### RQ1.

What is the impact of shortening fieldwork duration on response rates at three phases of fieldwork, corresponding to the launch of the study and early production, the continuation of main fieldwork, and the end of study? We implement a treatment duration that is identical to the control duration except during the middle phase of main fieldwork, which is shorter for the treatment group by 8weeks. We expect treatment cases to have a lower response rate during this phase only. By the end of fieldwork, we expect no difference in final response rates between the two groups. We expect that the adaptive end-of-study strategy, including higher incentives, will result in sufficient numbers of treatment interviews, offsetting the lower response rate during the abbreviated period of main production.

Based on our expectation of no difference in final response rates between the groups, we further predict that the sample composition of completed cases will not vary by experimental condition.

### RQ2.

What is the impact of the shorter duration on time to complete the interview, and on fieldwork effort, as assessed by the total number of interviewer attempts to contact respondents and encourage them to complete the interview? Given the shorter fieldwork duration of the treatment group, we expect the treatment cases to respond more quickly on average than control cases. We expect that the treatment group will have fewer interviewer contact attempts (telephone calls, and email and text message reminders) during the shortened middle phase of fieldwork which will result in fewer attempts overall compared to the control group.

### RQ3.

What are the cost implications of the shorter duration of fieldwork? We acknowledge some uncertainty regarding how the shorter duration will affect overall costs. On the one hand, we anticipate that similar response rates and quicker interview completion for the treatment group will lead to cost-savings due to reduced interviewer effort during the compressed fieldwork period. However, it is uncertain to what extent higher incentive costs might be required to motivate the treatment cases to complete the interview in a shorter timeframe, which could potentially offset the savings from reduced interviewer effort.

### RQ4.

Finally, how will quality of survey responses be affected by the shorter fieldwork duration? Based on our expectation of similar response rates between the two groups, we expect no differences in rates of item nonresponse or interview length.

## 3. DATA AND METHODS

### 3.1 Study design

The fieldwork duration experiment was embedded in the 2023 wave of the Panel Study of Income Dynamics, a nationally representative household panel study of US families (see McGonagle et al. 2012). The survey instrument collects a variety of information on economic, health, and social behavior. The study interviews one adult in each family, typically the individual who is most knowledgeable about the family finances. Over most waves of the study, families have been offered a post-paid, conditional monetary incentive to participate (i.e., US$80 in 2023). Additional monetary incentives are also offered to some respondents to encourage response, including higher incentives at the end of fieldwork. Fieldwork is conducted by a professionally trained staff employed by the University of Michigan. After many waves collecting data primarily through telephone interviews, in 2021, the study made a planned transition to a sequential mixed-mode design (web/CATI).

The 2023 wave followed 10,928 families, including 8,944 families who participated in the prior wave (“re-interviews”). Cases were released to the field in replicates between March and June 2023. All data collection activities were completed by December 31, 2023. Nearly 100 percent of re-interview families and approximately 80 percent of all families received an initial assignment to complete the interview on web. By the end of fieldwork, interviews were completed with 9,189 families for an overall response rate of 86 percent and a re-interview response rate of 94 percent (AAPOR RR6; The American Association for Public Opinion Research 2023).

### 3.2 Experimental design and fieldwork protocols

A total of 2,363 re-interview respondents were randomly selected for the experiment from the overall pool of 8,944 re-interview respondents, and an approximate random half of these cases were assigned to the treatment group (*N* = 1,179) and the remainder to the control group (*N* = 1,184; supplementary table S1). Respondents in both experimental groups were assigned to a web-first condition followed by an option to complete the interview by telephone after eight weeks of nonresponse. All respondents were administered the same survey instrument. The treatment group received accelerated fieldwork protocols over a 20-week field period, and the control group received the same protocols with the standard duration of a 28-week field period.

The choice of 20weeks as the treatment duration was an attempt to strike a balance between a shorter length that would yield meaningful cost savings compared to the 28-week standard duration, yet was also sufficient to achieve fieldwork goals. In other words, a duration of less than 20weeks would make it impractical to implement fieldwork protocols and could endanger response rate goals, and one that was longer (i.e., greater than 20weeks but less than 28weeks) might not provide meaningful cost savings.

Fieldwork protocols for both experimental conditions were implemented across three phases, corresponding to the launch of fieldwork, continuing fieldwork, and the fieldwork close-out (table 1). The duration of the phases at the beginning and the end of fieldwork (i.e., Phase 1 and Phase 3) was the same for both groups. The duration of the middle phase of fieldwork (Phase 2) was experimentally manipulated, with the treatment group receiving eight fewer weeks of fieldwork than the control group (6 weeks vs. 14 weeks). Activities within the phases were the same for both groups.

Phase 1, lasting eight weeks, began with the announcement of the launch of the upcoming wave of the study, via a letter sent through postal mail, and subsequent email and text messages sent over a three-week period. These initial messages provided respondents with information about the study, described the incentive that would be sent upon completion of the interview, and gave each respondent unique login credentials along with instructions on how to access the web-portal. This was followed by a five-week period during which interviewers were instructed to send one to two email or text messages per week, to respondents encouraging their participation. At week six, additional emails and texts were sent offering respondents an extra incentive ($20) if they completed the interview within the next two weeks (i.e., a time-limited incentive offer). Email and text messages were pre-written for interviewer use by the study staff and were reviewed and approved by the university Human Subjects Institutional Review Board.

During Phase 2, which started at the end of the two-week time-limited incentive offer, the incentive reverted to the baseline amount ($80), and interviewers began making at least one telephone call offering respondents the option of completing the interview by telephone, although respondents could also complete the interview by web. Interviewers continued to send one to two weekly email and text reminders. The main experimental intervention occurred during this phase, consisting of an abbreviated six-week period of contact attempts made by interviewers via telephone, email, and text for the treatment group compared to the full fourteen-week period for the control group. During Phase 3, final close-out activities were implemented across six weeks, including multiple weekly messages sent via email, text, and telephone that alerted respondents to the upcoming end of the study using a specific end-date and offered a notably higher incentive ($150–$200; the “endgame offer”).

Interviewers worked cases in both experimental conditions and across all other approximately 8,500 cases that were not involved in the experiment. They were trained to manage all of their active cases using sample management software and adhere to weekly fieldwork protocols that varied over time and by release. The staggered release of the total sample meant that the cases worked by interviewers had varying timelines, often with different contact protocols in a week. All cases except those in the treatment condition had an end date of December 31, which has been the established end date for many waves. Interviewers were not blind to the assignment of cases to the treatment condition for two main reasons. First, the treatment protocol used an earlier end date, necessitating interviewer awareness of the ending phase of the protocol. Second, interviewers may have been asked by families in the treatment group about the early end date, which was mentioned in the advance letter, and why their relatives in the study had a different timeline. Ultimately no such questions were raised. Because the release of cases was staggered, the assignment to the treatment condition was not salient to interviewers until Phase 3 began.

### 3.3 Outcome measures

We compare the following fieldwork outcomes for respondents randomized to the two experimental conditions: *response rates*, calculated as the percentage of eligible respondents completing an interview (based on definition RR6, The American Association for Public Opinion Research 2023); *the total number of interviewer contact attempts* that were expended to finalize an interview or by the end of fieldwork for nonresponse cases, constructed as the sum of emails, text messages, and telephone calls; and *fieldwork duration*, defined as the number of weeks from the beginning of fieldwork to the date when a final disposition status was assigned (i.e., either response or nonresponse). *Fieldwork costs* are calculated across all cases, and separately for completed cases and nonresponse cases, as the sum of costs for interview contact attempts and respondent incentives. *Interview quality* is examined using average and median item nonresponse rate, defined as the percentage of items skipped in web interview or answered “don’t know” in a telephone interview, and the mean and median number of interview minutes.

### 3.4 Demographic and socioeconomic measures

We examined a series of demographic and socioeconomic variables to confirm the randomization of respondents assigned to the treatment and control conditions and to evaluate treatment effects, including the following variables (1 = yes, 0 = no): female sex of respondent; age of respondent in continuous years; married or cohabiting versus single, widowed, or divorced; whether children reside in the household; total family income less than or equal to the median versus greater than the median; educational attainment, coded as less than 12years versus greater than 12years; metropolitan versus non-metropolitan residential location; and dummy variables to classify cases by their sample origin—original national probability sample, original low-income oversample, or an immigrant refresher sample.

### 3.5 Analysis strategy

We first confirm the randomization of cases assigned to each experimental condition by comparing their socio-demographic characteristics using cell frequencies and evaluating the difference with Wald Chi-square tests. Using *t*-tests, we evaluate mean differences between the experimental conditions in fieldwork outcomes, including response rates, the total number of interviewer contact attempts, rates of item nonresponse, and interview minutes. We use logistic regression to test overall differences between the experimental conditions in sample composition among completed interviews. We also test differences between the treatment and control groups in time-to-completion for the interview (across the entire field period and by fieldwork phase) using Kaplan–Meier non-parametric survival analysis (Kaplan and Meier 1958).

We compare the major costs of the experimental conditions by evaluating the two main fieldwork cost-drivers for each experimental condition: interviewer effort in the form of contact attempts for interview cases and nonresponse cases, and incentive costs for cases completing the interview. Cost estimates for total interviewer contact attempts are calculated by multiplying the number of cases and estimated costs per attempt by the average number of total contact attempts, separately for completed interviews and nonresponse cases. Cost estimates for interviewer contact attempts are based on actual wages and fringe benefits. We assume that each attempt type requires the same amount of interviewer time, including reviewing notes about prior interactions with the respondent, typing or applying a template to send an email or text message, and dialling and speaking directly with a respondent or leaving a voicemail message. We provide incentive costs for completed interviews only since nonresponse cases have zero incentive costs. Grand total costs are generated by summing the costs for interviewer attempts and incentives.

Additional details regarding the analyses of the complex survey design are reported in the Preferred Reporting Items for Complex Survey Sample Analysis figure (PRICSSA; supplementary figure S1).

## 4. RESULTS

### 4.1 Response rates and sample composition

Table 2 compares response rates by experimental condition across three phases of fieldwork: Phase 1, from the start of fieldwork to the end of the week 8 time-limited offer, Phase 2, regular fieldwork lasting six weeks for the treatment group and 14 weeks for the control group, and Phase 3, the 6-week study close-out period. As would be expected due to the use of identical protocols during Phase 1, response rates for the two groups are the same after the first eight weeks of fieldwork (59.3 percent and 59.0 percent, *p* = NS). During Phase 2, the cumulative, unconditional response rate of the control group exceeds the treatment group by nearly 7 percentage points (73.1 percent vs. 66.5 percent, respectively, *p* = .03). By the end of fieldwork (Phase 3), and unexpectedly, the final response rate of the treatment group exceeds the control group by 2.8 percentage points (95.0 percent vs. 92.2 percent, respectively, *p* = .006).

The overall test of significance for a logistic regression comparing the observed characteristics of the two experimental groups among completed interviews is non-significant (Wald *χ*^2^ = 0.84(11), *p* = NS; supplementary table S3).

The intervention’s accelerated protocols changed the timing of interview completion, as highlighted by the results for conditional response rates (i.e., the proportion responding in each phase among cases still active) during Phase 2 and Phase 3. With nearly identical completion rates in Phase 1, both groups entered Phase 2 with approximately the same number of cases. During Phase 2—following the expiration of the time-limited incentive and before the end-of-study higher incentive offer—about twice as many control cases completed the interview as treatment cases (34.6 percent vs. 17.7 percent, *p* ≤ .0001), an unsurprising result given the shorter duration of this phase for the treatment cases. This in turn led to a comparatively greater share of treatment cases to be eligible for Phase 3, and a higher completion rate in this final phase compared to the control group (85.1 percent vs. 71.1 percent, *p* ≤ .0001).

### 4.2 Time to Interview Completion

**Figure 1.**
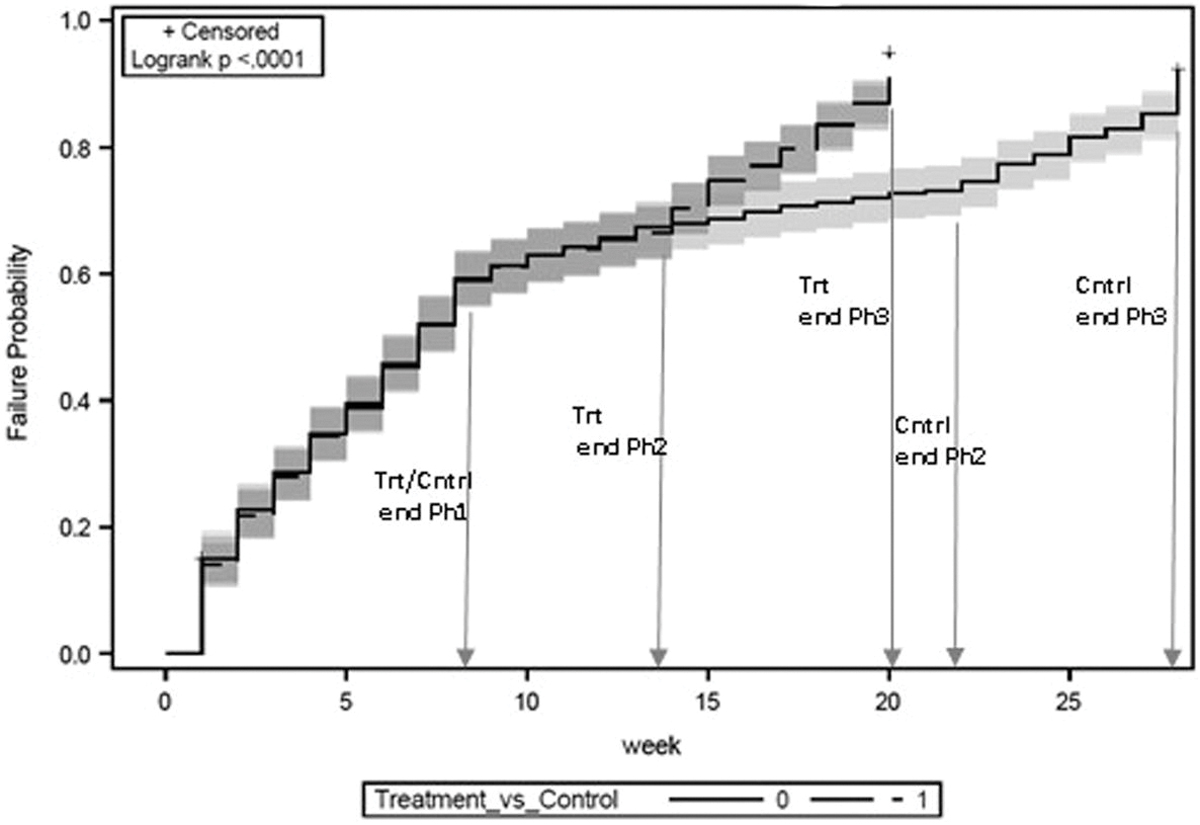
Time to Interview Completion by Experimental Condition.

By the end of fieldwork, the accelerated fieldwork protocols led to the treatment cases being completed at a faster rate than the control group, as indicated by the statistical significance of the test of differences in Kaplan–Meier curves by experimental condition (figure 1; log-rank test *χ*^2^ = 63.1(1), *p* ≤ .0001). Examination of the time to interview completion by the three phases of fieldwork shows that the pace of completion for the two groups was the same through the first eight weeks of fieldwork—that is, Phase 1—when the protocols were identical (log-rank test *χ*^2^ = 0.004(1), *p* = NS). During Phase 2, which marked the implementation of the intervention to shorten fieldwork duration, the pace of completion of the groups remained the same until the terminus of the phase for the treatment cases—as indicated by the overlapping curves through week 13. Following this, the control cases had an additional eight weeks of fieldwork, as indicated by the non-overlapping curve in the figure (log-rank test *χ*^2^ = 19.5(1), *p* ≤ .0001). Finally, during Phase 3, fieldwork for the treatment group has a faster and higher completion rate as indicated by the steeper curve compared to the control group (log-rank test *χ*^2^ = 503.4(1), *p* ≤ .0001).

### 4.3 Interviewer Effort

**Figure 2.**
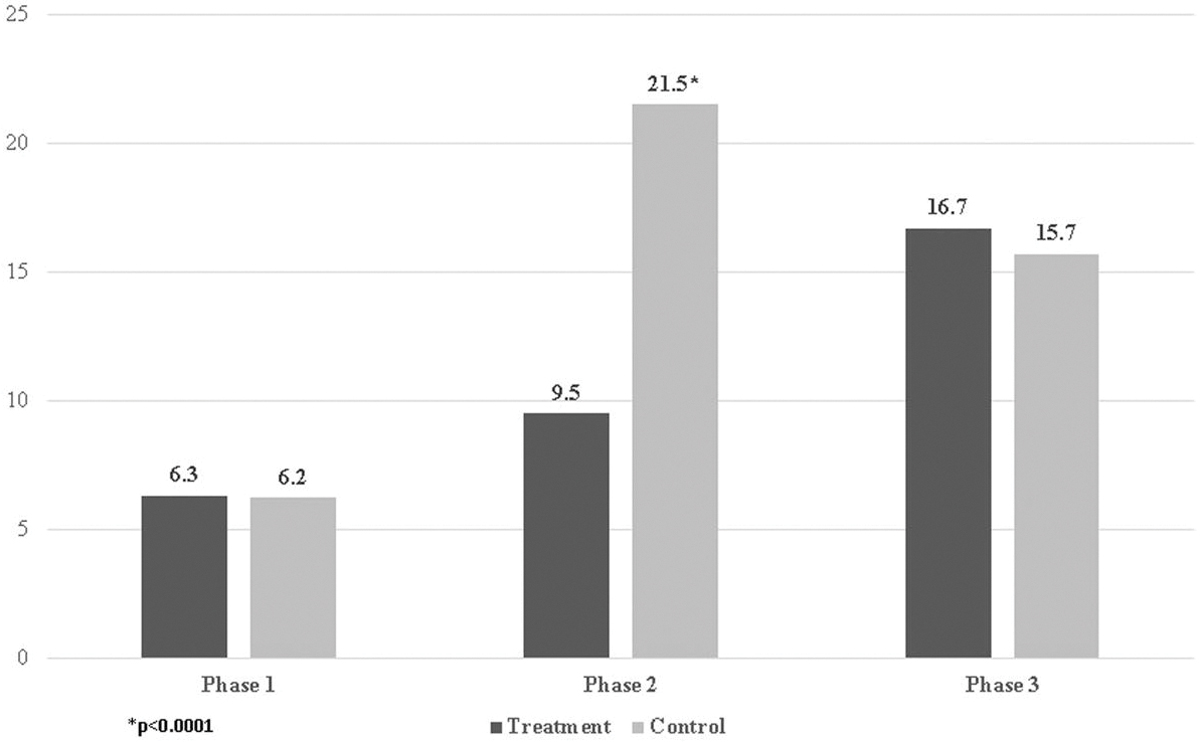
Contact Attempts (Mean) Among Cases Still Active in Each Phase.

We examined the total number of interviewer contact attempts by experimental condition overall and during each phase. Across all weeks of fieldwork, the shortened duration yielded significantly fewer average contact attempts compared to the standard protocol. The treatment cases received an average of 15.8 attempts compared to 19.3 for the control cases (3.5 fewer attempts, *p* ≤ .0001), a reduction of more than 18 percent. As shown in figure 2, the source of the reduction is due to the dramatically fewer attempts made to the treatment cases during Phase 2 compared to the control cases (9.5 vs. 21.5 mean attempts (12 fewer attempts, *p* ≤ .0001) for the treatment vs. control groups, respectively), a reduction of more than 50 percent. As expected, there was no difference between the groups in the number of attempts in either Phase 1 or Phase 3—during which time the protocols were equivalent—among cases still active at the start of these phases.

In sum, the accelerated fieldwork protocol had lower overall interviewer effort. The higher overall average number of attempts in the control group can be traced to the additional time in regular fieldwork during Phase 2 when these cases received an average of 12 additional attempts.

### 4.4 Cost Evaluation

We evaluated potential cost savings of the intervention by comparing the two main fieldwork cost-drivers for each experimental condition: interviewer effort in the form of contact attempts for interview cases and nonresponse cases, and incentive costs for cases completing the interview.

On an overall cost-per-case basis, we found that the treatment cases were less expensive than the control cases by $15 per case (table 3; grand total cost per case difference), a percentage difference of 7.1 percent. For cases completing the interview, the accelerated treatment protocols yielded average cost savings of $17 per case compared to the standard protocols; however, the treatment incentive costs were higher by an average of $8 per case, somewhat offsetting these savings. The higher incentive costs for the treatment group were due to a higher overall response rate and less relative time compared to the control group in regular fieldwork when the baseline incentive was being offered and more relative time during the end-of-study period when the higher incentive was being offered. For the small share of nonresponse cases, the treatment saved an average of $46 per case, as the accelerated protocols resulted in a lower number of nonresponse cases, and a shorter period of time to make contact attempts to the cases compared to the standard protocols.

### 4.5 Interview Quality

We found no differences between the experimental conditions in rates of item nonresponse or interview length (table 4). On average, respondents in both groups skipped or declined to answer about 5 percent of all items (about 4 percent at the median; *p* = NS for both mean and median comparisons). The mean interview length was 117.3 for treatment cases (102.6 at the median) and 118.4 for control cases (104.3 at the median; *p* = NS for both mean and median comparisons).

## 5. DISCUSSION AND CONCLUSIONS

Within the framework of a mixed-mode design for an ongoing household panel survey, we provide empirical evidence that fieldwork goals can be achieved, and may even be improved, by shortening the data collection period. The experimental intervention to accelerate data collection was highly successful, yielding significantly higher response rates, as well as cost-savings due to less interviewer effort and a shorter field period, with no final differences in sample composition or decrements in response quality. The response rate results were especially noteworthy as the final response rates of the treatment group unexpectedly surpassed those of the control group.

There are several possible reasons for this finding. One potential explanation is that the changing case mix due to the shorter duration of the treatment during Phase 2 (i.e., fewer treatment cases completed the interview during middle production) led to a higher share of cooperative cases who were offered and responsive to the end-of-study protocol (i.e., during Phase 3) in the treatment group than in the control group. The end-of-study protocol included both a substantially higher incentive and messages that highlighted the imminent “end of the study” along with a final end date. In contrast, the additional time in Phase 2 led to a larger number of control cases completing the interview during this phase, and those still active by Phase 3 were less cooperative and/or harder to contact and hence were less responsive to the higher incentives and time-delimited appeals.

Various social psychological effects due to the shortened field period may have also prompted greater cooperation by the treatment cases. For example, consistent with leverage-saliency theory (Groves et al. 2000), the novelty of the significantly earlier end date as highlighted in reminder messages to treatment cases, along with the perceived benefits of participation, including a higher end-of-study incentive, may have captured more attention than messages describing the usual end date of the standard protocol. Moreover, consistent with the scarcity principle (e.g., Cialdini 1993), the shortened deadline may have elicited a more prompt response from treatment cases who had less time to capitalize on the perceived benefits of completing the interview. Our respondent reminders deliberately emphasized the approaching end date with messaging intended to create a sense of urgency, motivating individuals to respond to avoid regret (e.g., Swain et al. 2006; Pieters and Zeelenberg 2007; Zeelenberg and Pieters 2007). A question for future research is whether the observed benefits of the shorter protocol will persist across subsequent waves in a panel context, given that its initial novelty might fade over time.

We also examined the possibility that treatment response rates were higher because interviewers prioritized the accelerated protocol and devoted more contact attempts to the treatment cases. However, the results of the analysis of interviewer contact attempts show that interviewers made the required number of attempts during the different phases of fieldwork, and—as we expected—treatment cases had significantly fewer overall attempts than control cases. Treatment cases received fewer overall interviewer attempts, attributable to the shortened field period; during the treatment end-of-study phase (Phase 3), control cases continued to receive ongoing interviewer attempts as part of standard fieldwork protocols, but these yielded relatively few completed cases. Overall, the extra eight weeks of fieldwork of the standard protocol were comparatively inefficient, yielding a lower response rate and more interviewer contact attempts compared to the accelerated protocol.

As a consequence of achieving a higher response rate with fewer interviewer resources over a shorter field period, the intervention saved costs. One source of uncertainty in the implementation of the treatment intervention was whether or not the potential increase in incentive payments due to the shortened field period (i.e., a higher number of cases were eligible for the increased incentive at the end of fieldwork) would be offset by a reduction in interviewer effort. We found that while incentive costs were in fact modestly higher for the treatment group, they were more than offset by cost savings due to fewer interviewer attempts and the higher response rate of this group. With the protracted field period of the control group, interviewer costs for the nonresponse cases in this group were especially high.

We believe that the current study provides the first experimental evaluation of the effects of shortening the length of fieldwork on data collection outcomes in a mixed-mode panel study. A key strength is the random assignment of respondents to either the shorter treatment duration or the standard duration, with the experiment embedded in a nationally representative household panel study. However, there are several limitations that should be considered when interpreting the results.

First, interviewers were not blind to the assignment of cases to the treatment condition. This was unavoidable due to the need for interviewers to correctly apply close-out protocols early for the treatment group. Although the assignment of cases to the treatment should not have been salient to interviewers until close-out activities began—because interviewers had a mix of cases with different weekly protocols across both experimental groups and from other releases—it is possible that interviewers devoted more attention to the treatment cases. However, as we described, the results showed that interviewers adhered to the protocols and did not make more contact attempts to treatment cases during the initial phase of fieldwork, during the initial period of Phase 2, in which the protocol was identical, or during the endgame. Moreover, messages sent to respondents were all drawn from pre-written templates, mitigating the chances that treatment cases received differential or “higher quality” messages. Future research should attempt to design an experiment that facilitates the application of fieldwork protocols in a “blind” setting, for instance, by having both treatment and control groups receive identical endgame protocols through a later fieldwork start for the treatment group.

Second, the study assessed only one alternative fieldwork duration compared to the usual duration, which limits our understanding of how other potential durations may affect fieldwork goals. That is, the external validity of the current study is limited to the specific duration that was examined, and the use of different fieldwork durations may lead to varying outcomes. To further optimize the balance between survey costs and field outcomes, additional experimental manipulations of fieldwork duration could be examined. For example, alternate designs might assess the impact of offering higher incentives earlier in fieldwork to encourage quicker interview completion. Previous experimental studies have shown that providing high incentives early can lead to rapid interview completion and cost-efficiency, particularly with challenging or resource-intensive cases (Fomby, Sastry and McGonagle 2017; McGonagle et al. 2023). However, this approach may result in increased incentive costs by “over-incentivizing” respondents who may be willing to accept lower incentives for prompt participation. Another design could evaluate extending the middle phase of data collection, which could potentially reduce overall costs by allowing more cooperative cases to complete the interview before the application of higher incentives at the end of fieldwork, although this may increase field management costs. Finding the optimal balance between survey duration and meeting data collection objectives is a major challenge for survey practitioners.

Third, the experiment was limited to respondents who participated in the prior wave, leaving the effects of an accelerated field period on non-respondents and new participants unexplored. An accelerated protocol may be less effective for these groups as they might require more time for contact and encouragement; however, it is also possible that such benefits could extend to them as well. This issue warrants further investigation in future research.

Fourth, the overall generalizability of the current study to other panel studies may be limited by differences in design features, such as the use of endgame strategies, including higher incentives and respondent messaging designed to emphasize the study deadline, the availability of interviewers, characteristics of the target population, as well as the effects of seasonality, prevailing secular environments, and other external factors. These variations may affect the extent to which the results can be applied to other survey contexts.

Finally, the cost-savings model provides a lower bound estimate for the accelerated protocol as there are additional savings in field operation management costs associated with a shorter active fieldwork period that are not possible to quantify in the current study. Future evaluations should aim to independently assess management costs for field periods of varying durations. Although challenging, this would provide a more comprehensive understanding of the potential cost implications associated with shortening fieldwork length.

Despite these limitations, our findings underscore the significant role of the duration of fieldwork as a key survey design feature that can be strategically leveraged to optimize data collection outcomes in a mixed-mode environment.

## Supplementary Material

Supplemental materials

## Figures and Tables

**Table 1.** Number of Weeks by Fieldwork Phase and Experimental Condition

Phase	Treatment	Control

**1. Start of fieldwork to end of time-delimited offer**	8	8
**2. Continuing fieldwork, telephone offered**	6	14
**3. End-game incentive offer to study end**	6	6
**Total**	20	28

**Table 2.** Response Rates by Fieldwork Phase by Experimental Condition

	Treatment group			Control group			Difference	
	No. of cases	No. of completed interviews	RR (%)	No. of cases	No. of completed interviews	RR (%)	RR % Δ	*p* of Δ

**Cumulative (unconditional) RRs by end of phase**								
Phase 1	1,179	699	59.3	1,184	698	59.0	0.3	.86
Phase 2	1,179	784	66.5	1,184	866	73.1	−6.6	.03
Phase 3	1,179	1,120	95.0	1,184	1,092	92.2	2.8	.006
**RRs during each phase**								
***Incremental RRs—among all cases***								
Phase 1	1,179	699	59.3	1,184	698	59.0	0.3	.86
Phase 2	1,179	85	7.2	1,184	168	14.2	−7.0	<.0001
Phase 3	1,179	336	28.5	1,184	226	19.1	9.4	<.0001
***Conditional RRs—among those still active in each phase***								
Phase 1	1,179	699	59.3	1,184	698	59.0	0.3	.86
Phase 2	480	85	17.7	486	168	34.6	16.9	<.0001
Phase 3	395	336	85.1	318	226	71.1	14.0	<.0001

**Table 3.** Cost evaluation of intervention

	Treatment	Control	Difference

Number of cases	1179	1184	
Average cost per interviewer attempt	$7.25		
Costs based on interviewed cases	1,120	1,092	
***1. Interviewer contact attempts***			
Interviewer attempts (mean)	14.1	16.4	−2.3
Total cost	$114,492	$129,839	$(15,347)
Total cost per case	$102	$119	$(17)
***2. Incentive costs***			
Main fieldwork offer ($80–100)	$67,450	$74,210	$(6,760)
Endgame offer ($150–200)	$59,200	$40,750	$18,450
Total cost	$126,650	$114,960	$11,690
Total cost per case, based on interview cases	$113	$105	$8
Costs based on nonresponse cases	59	92	
***1. Interviewer contact attempts***			
Interviewer attempts (mean)	47.3	53.6	−6.3
Total cost	$20,233	$35,751	$(15,519)
Total cost per case	$343	$389	$(46)
***2. Incentive costs***	0	0	0
Grand total costs	$261,375	$280,550	$(19,175)
Grand total costs per case	$222	$237	$(15)

**Table 4.** Interview Quality Indicators

	Treatment *N* = 1,114	Control *N* = 1,089

Item missing data rate		
Mean	5.0%	5.1%
Median	4.1%	4.2%
Interview length		
Mean	117.3	118.4
Median	102.6	104.3

Note.—*N* = 6 treatment cases and *N* = 3 control cases were missing on the item missing data rate and Interview length.

## Data Availability

The de-identified data and code are available through the PSID OpenICPSR data repository (https://www.icpsr.umich.edu/sites/psid/home).

## Statement of Significance

Panel studies worldwide are implementing sequential mixed-mode designs, which prioritize the use of web, followed by options for telephone or in-person responses, as a strategy to maximize response rates and reduce survey costs. Interviews completed on the web are collected faster than interviewer-administered interviews, but few studies have examined fieldwork duration or evaluated the effect of the length of data collection on fieldwork outcomes. This article reports on findings from an experiment embedded in a long-running, nationally representative US household panel study designed to assess the impact of shortening the time over which data are collected on response rates, sample composition, interviewer effort, survey costs, and interview quality. Compared to the standard length of data collection, the experimental intervention aimed at accelerating data collection led to significantly higher response rates and cost savings. These benefits were achieved due to reduced interviewer effort and a shorter field period, all while maintaining response quality.
