# Supplementary material for: Meeting Data Collection Goals Quicker: An Experimental Evaluation to Reduce Fieldwork Duration in a Mixed-Mode Panel Study
Source: J Surv Stat Methodol. 2026 Jan 8;14(2):374–93. doi: 10.1093/jssam/smaf030 (PMC12970957; doi:10.1093/jssam/smaf030)
Supplement: smaf030_Supplementary_Data [file smaf030_supplementary_data.docx]

**Supplemental Materials**

Table S1. Randomization check of assignment to the experimental conditions

Comparisons between the cases assigned to the experimental conditions across socio-demographic respondent characteristics are shown in Table S1. We evaluated the difference between the two groups using t-tests to compare continuous variables and Chi-square tests to compare discrete variables. As would be expected due to random assignment, there were no statistically significant differences between the conditions across any of the respondent characteristics. The Wald chi-square test of the hypothesis that all coefficients in a logistic regression model comparing the two groups are 0 was confirmed, indicating that the groups are balanced across socio-demographic model covariates and random assignment to the experimental conditions was met.
