# Supplementary material for: MEETING DATA COLLECTION GOALS QUICKER: AN EXPERIMENTAL EVALUATION TO REDUCE FIELDWORK DURATION IN A MIXED-MODE PANEL STUDY
Source: J Surv Stat Methodol. Author manuscript; Available in PMC 2026 Mar 10. (PMC12970957; doi:10.1093/jssam/smaf030)
Supplement: Supplementary file 1 [file NIHMS2143631-supplement-Supplemental_materials.pdf]

Supplemental Materials

Table S1. Randomization check of assignment to the experimental conditions

Comparisons between the cases assigned to the experimental conditions across socio-demographic respondent characteristics are shown in Table S1. We evaluated the difference between the two groups using t-tests to compare continuous variables and Chi-square tests to compare discrete variables. As would be expected due to random assignment, there were no statistically significant differences between the conditions across any of the respondent characteristics. The Wald chi-square test of the hypothesis that all coefficients in a logistic regression model comparing the two groups are 0 was confirmed, indicating that the groups are balanced across socio-demographic model covariates and random assignment to the experimental conditions was met.

Table S1. Respondent Characteristics at Assignment By Experimental Condition

Respondent Characteristic	Treatment (N=1179)	Control (N=1184)	Test of Difference
Female Respondent (%)	62.2	60.7	$X^2(1)=0.52$, p=0.5
Years of Age of Respondent (Median)	43.0	42.0	t=-1.2, p=0.2
Married (%)	49.4	52.8	$X^2(1)=2.77$, p=.10
No Children (%)	60.3	60.4	$X^2(1)=0.01$, p=0.9
Family Income (Median)	$56,784	$60,310	t=1.1, p=0.3
Years of Education (Median)	14.0	14.0	t=0, p=1.0
Urban Residence (%)	84.8	84.1	$X^2(1)=0.22$, p=0.6
Sample Type (%): a) National probability sample, b) Low income oversample, c) Immigrant refresher	59.5, 35.4, 5.1	59.6, 34.1, 6.2	$X^2(2)=1.71$, p=0.4

Note: Wald chi-square test of logistic regression comparing the two groups was nonsignificant ($\chi2(9)=6.1$, p=0.7).

Table S2. Sample Composition by Experimental Condition Among Completed Interviews			
	Treatment group (vs. Control group)		
**Variables**	**Estimate**	**Standard Error**	**p**
Female respondent (vs. male)	0.02	0.09	0.82
Age of respondent	0.02	0.02	0.25
Age of respondent squared	-0.0001	<0.01	0.43
Married/cohabiting (vs. single, widowed, divorced)	-0.18	0.10	0.07
Zero children in family unit (vs. 1 or more)	-0.11	0.10	0.29
Metropolitan area of residence (vs. non-metropolitan)	-0.01	0.12	0.94
Total family income	4.12E-07	<0.01	0.53
Total family income squared	-588E-16	<0.01	0.74
Original oversample sample member (vs. national sample)	0.02	0.10	0.86
Immigrant refresher sample member (vs. national sample member)	-0.13	0.18	0.46
<12 years of education (vs. 12 years or more)	0.005	0.10	0.96
Intercept	-0.45	0.42	0.28
Wald $\chi2$ =0.84 (11), p<=0.60.			
N	2,212		

Note: Logistic regression model fits the data using the maximum likelihood method and ajusts for the stratified and clustered survey design; variances are computed using a Taylor series estimation.

Figure S1. Preferred Reporting Items for Complex Survey Analysis (PRICSSA) Checklist	
Name and wave of survey:	Panel Study of Income Dynamics, wave 43
Data collection mode:	sequential mixed-mode (web, computer-assisted telephone interviewing)
Dates of data collection:	June 19, 2023 - December 31, 2023
Target population:	nationally representative sample of U.S. households
Populations excluded:	active-duty military, incarcerated persons
Design:	multistage,stratified cluster sample
Variance estimation:	Taylor Series
Weight and design variables	
Weight:	None
Cluster:	Sample identification variable
Stratum:	Sample group membership
Unweighted total sample size:	2,363
Response rate:	93.6% (AAPOR RR6)
Software:	SAS statistical software
Location of code and data:	PSID OpenICPSR Data Repository
